# Circular sequence comparison: algorithms and applications

**DOI:** 10.1186/s13015-016-0076-6

**Published:** 2016-05-10

**Authors:** Roberto Grossi, Costas S. Iliopoulos, Robert Mercas, Nadia Pisanti, Solon P. Pissis, Ahmad Retha, Fatima Vayani

**Affiliations:** Department of Informatics, University of Pisa, Pisa, Italy; ERABLE team, INRA, Paris, France; Department of Informatics, King’s College London, London, UK; Department of Computer Science, Kiel University, Kiel, Germany

## Abstract

**Background:**

Sequence comparison is a fundamental step in many important tasks in bioinformatics; from phylogenetic reconstruction to the reconstruction of genomes. Traditional algorithms for measuring approximation in sequence comparison are based on the notions of distance or similarity, and are generally computed through sequence alignment techniques. As *circular* molecular structure is a common phenomenon in nature, a caveat of the adaptation of alignment techniques for circular sequence comparison is that they are computationally expensive, requiring from super-quadratic to cubic time in the length of the sequences.

**Results:**

In this paper, we introduce a new distance measure based on *q*-grams, and show how it can be applied effectively and computed efficiently for circular sequence comparison. Experimental results, using real DNA, RNA, and protein sequences as well as synthetic data, demonstrate orders-of-magnitude superiority of our approach in terms of efficiency, while maintaining an accuracy very competitive to the state of the art.

## Background

### Biological motivation

Circular molecular structures are present, in abundance, in all domains of life: bacteria, archaea, and eukaryotes; and in viruses. They can be composed of either amino or nucleic acids. The following is an overview of such occurrences, and exhaustive reviews can be found in [1] (proteins) and [2] (DNA).

Double-stranded, circular chromosomes and plasmids are found in most bacteria and archaea. Whole-genome comparison is a very useful tool in classifying bacterial strains, as well as inferring phylogenetic associations between them. This is due to the dense structure of bacterial chromosomes, caused by the absence of introns, and the organisation of genes into operons. The extended benefit of aligning plasmids is the ability to identify important genes, such as antibiotic resistance genes, thereby enabling their study and exploitation by genetic engineering techniques [3].

The most familiar examples of such structures in eukaryotes are mitochondrial (MtDNA) and plastid DNA. MtDNA is, in most cases, inherited solely from the mother, and so is generally conserved. Human MtDNA is double-stranded, with a length of 16,569 base pairs (bp), consisting of just 37 genes encoding 13 proteins and 24 RNA molecules [4]. The absence of recombination in these sequences allows them to be used as simple indicators of phylogenetic evolution, and their high mutation rate is a powerful discriminative feature [5, 6]. There also exist smaller structures, called extrachromosomal circular DNA, which are similar to plasmids in bacterial cells. They are described as one of the characteristics of genomic plasticity in eukaryotes [7] and may derive from MtDNA [8].

It is common knowledge that many viral genomes are circular. Viral genomes vary greatly in size and structure. They can be made up of either RNA or DNA, and can be single- or double-stranded. Multiple sequence alignment of viral genomes can be useful in the elucidation of novel sites of interest [9], as well as the inference of evolutionary relationships [10]. This is particularly important in studying their pathogenicity, due to the rapid rate of mutation of viruses. Viroids are plant pathogens that comprise very small, single-stranded, circular RNA. Their multiple sequence alignment could prove useful in the analysis of their secondary structures and, therefore, the mechanisms by which they infect host plant cells [11].

Naturally-occuring circular proteins are found in both prokaryotes and eukaryotes [1]. Bacteriocins are very small toxins produced by bacteria in order to compete with closely-related bacterial strains. Many of these are circular, including gassericin A, found in *Lactobacillus gasseri* LA39 [12], and circularin A, found in *Clostridium beijerinckii* [13]. An interesting phenomenon known to occur naturally in linear protein structures is circular permutation [14]. This can be exemplified by swaposins: proteins highly-similar to saposins, resulting from circularly permuted linear peptide sequences [15]. The ability to align linear sequences from circular proteins can significantly speed up and enhance their analyses, and could also lead to the discovery of novel pairs of circularly permuted proteins.

### Our problem

Conventional tools, designed for linear sequences, could yield an incorrectly high genetic distance between closely related circular sequences. Indeed, when sequencing molecules, the position where a circular sequence starts can be totally arbitrary. Due to this arbitrariness, a suitable rotation of one sequence would give much better results for a pairwise alignment, and hence highlight a similarity that any linear alignment would miss. A practical example of the benefit this can bring to sequence analysis is the following. Linearized human (NC_001807) and chimpanzee (NC_001643) MtDNA sequences, obtained from GenBank [16], do not start in the same region. Their pairwise sequence alignment using EMBOSS Needle [17] (default parameters) gives a similarity of 85.1 % and consists of 1195 gaps. However, taking different rotations of these sequences into account yields a much more significant alignment with a similarity of 91 % and only 77 gaps. This example motivates the design of efficient algorithms that are specifically devoted to the comparison of circular sequences [18–20].

In this paper, we consider the pairwise circular sequence comparison problem. Under the edit distance model, it consists in finding an optimal linear alignment of two circular strings. This problem, for two strings *x* and *y* of length *m* and $n \ge m$, respectively, can be solved under the edit distance model in time $\mathcal {O}(n m \log m)$ [21]. Several other super-quadratic [22] and *approximate* quadratic-time [23] algorithms exist. Trivially, for molecular biology applications, the same problem can be solved in time $\mathcal {O}(nm^2)$, if extending the problem with scoring matrices and affine gap penalty scores. A *direct* application of pairwise circular sequence comparison is progressive multiple circular sequence alignment [11, 24, 25]. Multiple circular sequence alignment has also been considered in [26] under the Hamming distance model.

To the best of our knowledge, there is no fast (that is, with sub-quadratic time complexity) and exact (or at least very accurate) algorithm for circular sequence comparison under some realistic model (that is, allowing *indels*). Taking into account edit distance rather than Hamming distance is computationally challenging as the search space for seeking similarity is wider. Algorithms that speed up the process of string matching, by filtering out candidate positions in which a particular string can never occur, are known as filters. Filters that work for Hamming distance do not work in general for edit distance [27] as well. An exception to this are the *q*-gram filtering techniques [28] that have successfully been used for string matching under the edit distance model (e.g. [29–31]), as well as for multiple local alignments, both under the Hamming [32] and edit [31] distance models.

### Our contribution

We present new efficient *q*-gram-based methods for pairwise circular sequence comparison. Specifically, our contribution is threefold.

1. We introduce the $\beta$*-blockwiseq-gram distance* between two strings *x* and *y*, that is, a more powerful generalization of the *q*-gram distance introduced as a string distance measure in [28]. Intuitively, and similarly to [29–31], this generalization comprises partitioning *x* and *y* in $\beta$*blocks* each, as evenly as possible, computing the *q*-gram distance between the corresponding block pairs, and then summing up the distances computed blockwise.
2. We present an algorithm based on the suffix array [33] that finds the rotation of *x* such that the $\beta$-blockwise *q*-gram distance between the rotated *x* and *y* is minimal, in time and space $\mathcal {O}(\beta m + n)$, where $m=|x|$ and $n=|y|$, thereby solving *exactly* the circular sequence comparison problem under the $\beta$-blockwise *q*-gram distance measure. We also present a simple heuristic algorithm to solve an *approximate* version of the problem.
3. We present an experimental study, using real and synthetic data, which demonstrates orders-of-magnitude superiority of our approach, in terms of efficiency, while maintaining an accuracy very competitive to the *optimal* obtained after considering all rotations of *x* against *y* using EMBOSS Needle.

The paper is organized as follows. "Definitions and properties" section gives some preliminary definitions, notation, and properties. "Algorithms" section describes two algorithms, one is a heuristic approach and the other is an exact algorithm for circular sequence comparison under the $\beta$-blockwise *q*-gram distance measure. "Implementation" section provides details of the implementation of the algorithms. "Experimental results" section presents the experimental results of the performance and accuracy of the algorithms. Finally, "Conclusions" section gives some concluding remarks and future proposals. A preliminary version describing a subset of the results in this paper appeared in [34].

## Definitions and properties

We begin with a few definitions, following [35]. We think of a *stringx* of *lengthm* as an array $x[0\mathinner {.\,.}m-1]$, where every *x*[*i*], $0 \le i < m$, is a *letter* drawn from some fixed *alphabet*$\Sigma$ of size $|\Sigma | = \mathcal {O}(1)$. We refer to any string $x \in \Sigma ^q$ as a *q-gram*. The *empty string* of length 0 is denoted by $\varepsilon$. A string *x* is a *factor* of a string *y* if there exist two strings *u* and *v*, such that $y=uxv$. Let *x* be a non-empty string and *y* be a string. We say that there is an *occurrence* of *x* in *y*, or, simply, that *xoccurs iny*, when *x* is a factor of *y*. The *Parikh vector* associated with a string $w\in \Sigma ^*$ is denoted by $\mathcal {P}(w)$ and represents a vector of size $|\Sigma |$, where each component denotes the number of occurrences in *w* of the corresponding letter from $\Sigma$.

Consider the strings *x*, *y*, *u*, and *v*, such that $y=uxv$. If $u=\varepsilon$, then *x* is a *prefix* of *y*. If $v=\varepsilon$, then *x* is a *suffix* of *y*. We denote by SA the *suffix array* of *y* of length *n*, that is, an integer array of size *n* storing the starting positions of all lexicographically sorted suffixes of *y*, i.e. for all $1 \le r < n$, we have $y[\textsf {SA}{}[r-1] \mathinner {.\,.}n-1] < y[\textsf {SA}{}[r] \mathinner {.\,.}n - 1]$ [33]. Let lcp (*r*, *s*) denote the length of the longest common prefix between $y[\textsf {SA}{}[r] \mathinner {.\,.}n - 1]$ and $y[\textsf {SA}{}[s] \mathinner {.\,.}n - 1]$, for all positions *r*, *s* on *y*, and 0 if they do not have a common prefix. We denote by LCP the *longest common prefix* array of *y* defined by LCP$[r]=\textsf {lcp}{}(r-1, r)$, for all $1 \le r < n$, and LCP$[0] = 0$. The inverse iSA of the array SA is defined by $\textsf {iSA}{}[\textsf {SA}{}[r]] = r$, for all $0 \le r < n$. SA, iSA, and LCP of *y* can be computed in $\mathcal {O}(n)$ time and space [36].

A circular string of length *m* can be viewed as a traditional linear string which has the left- and right-most letters wrapped around and glued together in some way. Under this notion, the same circular string can be seen as *m* different linear strings, which would all be considered equivalent. Given a string *x* of length *m*, we denote by $x^{i}=x[i \mathinner {.\,.}m-1]x[0 \mathinner {.\,.}i-1]$, $0< i < m$, the *i*th *rotation* of *x* and $x^{0}=x$. For instance, the string $x=x^{0}=\mathtt {abababbc}$ has the following rotations: $x^{1}=\mathtt {bababbca}$, $x^{2}=\mathtt {ababbcab}$, and so on.

We give some further definitions following [28]. The *q-gram profile* of a string *x* is the vector $G_q(x)$, where $q > 0$ and $G_q(x)[v]$ denotes the total number of occurrences of *q*-gram $v \in \Sigma ^q$ in *x*. The *q*-gram distance between two strings *x* and *y* is defined as

1 $$\begin{aligned} D_{q}(x, y) = \sum \limits _{v \in \Sigma ^{q}} \left| G_q(x)[v] - G_q(y)[v] \right| . \end{aligned}$$

Note that $D_{q}$ is a *pseudo-metric* as $D_{q}(x, y)$ can be 0 even if $x \ne y$. $D_{q}$ has the following properties [28] for all $x, y, z \in \Sigma ^{*}$ of length at least *q*.

1. Positivity: $$D_{q}(x, y) \ge 0$$
2. Symmetry: $$D_{q}(x, y) = D_{q}(y, x)$$
3. Triangular inequality: $$D_{q}(x, y) \le D_{q}(x, z) + D_{q}(z, y)$$
4. $$\vert ( \vert x \vert - \vert y \vert ) \vert \le D_{q}(x, y) \le \vert x \vert + \vert y \vert -2q -2$$
5. $$D_{q}(x_{1}x_{2}, y_{1}y_{2}) \le D_{q}(x_{1},y_{1}) + D_{q}(x_{2},y_{2}) + 2(q-1)$$, for $$x_1, x_2, y_1, y_2 \in \Sigma ^{*}$$
6. $D_{q}(h(x), h(y)) \le D_{q}(x,y)$, for a non-length-increasing morphism *h* on $\Sigma ^*$.

### *Example 1*

Let $x=\texttt {GGAGTCTA}$, $y=\texttt {TTCTAGCG}$, and $q=3$. Table 1 shows the *q*-gram profiles of strings *x* and *y* and the *q*-gram distance between them. Each row represents the frequency of a *q*-gram in the given string. For succinctness of presentation, only those rows with frequency greater than zero (in either string) are shown, as well as rows representing $\texttt {AAA}$, $\texttt {CCC}$, $\texttt {GGG}$, and $\texttt {TTT}$ as points of reference.

**Table 1:** *q*-gram profiles of strings *x* and *y* and *q*-gram distance $D_{q}(x, y) = 8$ between them

(a) $$G_q(x)$$	
$\texttt {AAA}$	0
$\texttt {AGC}$	0
$\texttt {AGT}$	1
$\texttt {CCC}$	0
$\texttt {CTA}$	1
$\texttt {GAG}$	1
$\texttt {GCG}$	0
$\texttt {GGA}$	1
$\texttt {GGG}$	0
$\texttt {GTC}$	1
$\texttt {TAG}$	0
$\texttt {TCT}$	1
$\texttt {TTC}$	0
$\texttt {TTT}$	0
(b) $$G_q(y)$$	
$\texttt {AAA}$	0
$\texttt {AGC}$	1
$\texttt {AGT}$	0
$\texttt {CCC}$	0
$\texttt {CTA}$	1
$\texttt {GAG}$	0
$\texttt {GCG}$	1
$\texttt {GGA}$	0
$\texttt {GGG}$	0
$\texttt {GTC}$	0
$\texttt {TAG}$	1
$\texttt {TCT}$	1
$\texttt {TTC}$	1
$\texttt {TTT}$	0
(c) $$D_{q}(x, y)$$	
$\texttt {AAA}$	0
$\texttt {AGC}$	1
$\texttt {AGT}$	1
$\texttt {CCC}$	0
$\texttt {CTA}$	0
$\texttt {GAG}$	1
$\texttt {GCG}$	1
$\texttt {GGA}$	1
$\texttt {GGG}$	0
$\texttt {GTC}$	1
$\texttt {TAG}$	1
$\texttt {TCT}$	0
$\texttt {TTC}$	1
$\texttt {TTT}$	0

For a given integer parameter $\beta \ge 1$, we define a generalization of the *q*-gram distance in (1) by partitioning *x* and *y* in $\beta$*blocks* as evenly as possible, and computing the *q*-gram distance between each pair of blocks, one from *x* and one from *y*. The rationale is to enforce *locality* in the resulting overall distance. For the sake of presentation in the rest of the paper, we assume that the lengths $|x| = m$ and $|y| = n$ are both multiples of $\beta$, so that *x* and *y* are conceptually partitioned into $\beta$ blocks, each of size $m/\beta$ for *x* and $n/\beta$ for *y*.

### **Definition 1**

Given strings *x* of length *m* and *y* of length $n \ge m$ and integers $\beta \ge 1$ and $q > 0$, the $\beta$*-blockwiseq-gram distance*$D_{\beta ,q}(x, y)$ is defined as

2 $$\begin{aligned} D_{\beta ,q}(x, y)=\sum _{j=0}^{\beta -1}D_{q}\left( x\left[ \frac{jm}{\beta } \mathinner {.\,.}\frac{(j+1)m}{\beta }-1\right] , y\left[ \frac{jn}{\beta } \mathinner {.\,.}\frac{(j+1)n}{\beta }-1\right] \right) . \end{aligned}$$

### *Example 2*

Following Example 1, let $x=\texttt {GGAGTCTA}$ and $y=\texttt {TTCTAGCG}$, $q=3$, and $\beta = 2$. Further let $x_1=\texttt {GGAG}$, $x_2=\texttt {TCTA}$ and $y_1=\texttt {TTCT}$, $y_2=\texttt {AGCG}$ be the two blocks of *x* and *y*, respectively. Table 2 shows the *q*-gram profiles of strings $x_1$, $x_2$, $y_1$, and $y_2$; and the *q*-gram distance between $x_1$ and $y_1$ and the *q*-gram distance between $x_2$ and $y_2$.

**Table 2:** *q*-gram profiles of strings $x_1$, $x_2$, $y_1$, and $y_2$; *q*-gram distance between $x_1$ and $y_1$; and *q*-gram distance between $x_2$ and $y_2$, giving $D_{\beta ,q}(x, y) = 8$

(a) $$G_q(x_1)$$	
$\texttt {AAA}$	0
$\texttt {AGC}$	0
$\texttt {AGT}$	0
$\texttt {CCC}$	0
$\texttt {CTA}$	0
$\texttt {GAG}$	1
$\texttt {GCG}$	0
$\texttt {GGA}$	1
$\texttt {GGG}$	0
$\texttt {GTC}$	0
$\texttt {TAG}$	0
$\texttt {TCT}$	0
$\texttt {TTC}$	0
$\texttt {TTT}$	0
(b) $$G_q(y_1)$$	
$\texttt {AAA}$	0
$\texttt {AGC}$	0
$\texttt {AGT}$	0
$\texttt {CCC}$	0
$\texttt {CTA}$	0
$\texttt {GAG}$	0
$\texttt {GCG}$	0
$\texttt {GGA}$	0
$\texttt {GGG}$	0
$\texttt {GTC}$	0
$\texttt {TAG}$	0
$\texttt {TCT}$	1
$\texttt {TTC}$	1
$\texttt {TTT}$	0
(c) $$D_{q}(x_1, y_1)$$	
$\texttt {AAA}$	0
$\texttt {AGC}$	0
$\texttt {AGT}$	0
$\texttt {CCC}$	0
$\texttt {CTA}$	0
$\texttt {GAG}$	1
$\texttt {GCG}$	0
$\texttt {GGA}$	1
$\texttt {GGG}$	0
$\texttt {GTC}$	0
$\texttt {TAG}$	0
$\texttt {TCT}$	1
$\texttt {TTC}$	1
$\texttt {TTT}$	0
(d) $$G_q(x_2)$$	
$\texttt {AAA}$	0
$\texttt {AGC}$	0
$\texttt {AGT}$	0
$\texttt {CCC}$	0
$\texttt {CTA}$	1
$\texttt {GAG}$	0
$\texttt {GCG}$	0
$\texttt {GGA}$	0
$\texttt {GGG}$	0
$\texttt {GTC}$	0
$\texttt {TAG}$	0
$\texttt {TCT}$	1
$\texttt {TTC}$	0
$\texttt {TTT}$	0
(e) $$G_q(y_2)$$	
$\texttt {AAA}$	0
$\texttt {AGC}$	1
$\texttt {AGT}$	0
$\texttt {CCC}$	0
$\texttt {CTA}$	0
$\texttt {GAG}$	0
$\texttt {GCG}$	1
$\texttt {GGA}$	0
$\texttt {GGG}$	0
$\texttt {GTC}$	0
$\texttt {TAG}$	0
$\texttt {TCT}$	0
$\texttt {TTC}$	0
$\texttt {TTT}$	0
(f) $$D_{q}(x_2, y_2)$$	
$\texttt {AAA}$	0
$\texttt {AGC}$	1
$\texttt {AGT}$	0
$\texttt {CCC}$	0
$\texttt {CTA}$	1
$\texttt {GAG}$	0
$\texttt {GCG}$	1
$\texttt {GGA}$	0
$\texttt {GGG}$	0
$\texttt {GTC}$	0
$\texttt {TAG}$	0
$\texttt {TCT}$	1
$\texttt {TTC}$	0
$\texttt {TTT}$	0

In this paper, we consider the following problem, where we search for the *i*th rotation of *x* that minimizes its blockwise distance from *y* as defined in (2). Ties are broken arbitrarily.

Circular Sequence Comparison (CSC)

**Input:** strings *x* and *y* of lengths *m* and $n \ge m$, respectively, and integers $\beta \ge 1$ and $q < m$

**Output:***i* such that $D_{\beta ,q}(x^i, y)$ is minimal

## Algorithms

We use the following result to first give a naïve solution to the CSC problem.

### **Lemma 1**

[28]* If we have space *$\mathcal {O}(|\Sigma |^q)$* available, then theq-gram distance *$D_{q}(x, y)$* can be computed in time *$\mathcal {O}(m + n)$* and extra space *$\mathcal {O}(m + n)$*, where *$m=|x|$* and *$n=|y|$.

We then apply Lemma 1 to each pair of blocks of *x* and *y* separately.

### **Lemma 2**

*If we have space *$\mathcal {O}(|\Sigma |^q)$* available, then the*$\beta$*-blockwiseq-gram distance *$D_{\beta ,q}(x, y)$* can be computed in time *$\mathcal {O}(m + n)$* and extra space *$\mathcal {O}(\frac{m + n}{\beta })$*, where *$m=|x|$* and *$n=|y|$.

The naïve algorithm, denoted by nCSC, computes for $x'=xx$ the values

$$\begin{aligned} \delta _i=D_{\beta ,q}(x'[i \mathinner {.\,.}i + m - 1], y), \end{aligned}$$

for all $0 \le i <m$; we report position *i* such that $\delta _i$ is minimal. This requires the application of Lemma 2, *m* times. Therefore, we obtain the following.

### **Lemma 3**

*If we have space *$\mathcal {O}(|\Sigma |^q)$* available, then algorithm *nCSC* solves the CSC problem in time*$\mathcal {O}(m(m + n))$* and extra space *$\mathcal {O}(\frac{m + n}{\beta })$.

### Algorithm hCSC: a Heuristic algorithm

Here we give a simple heuristic algorithm, denoted by hCSC, to solve the CSC problem faster than nCSC, and return an approximation of the best rotation.

**Step 1**: We split $x'=xx$ in $2\beta$ non-overlapping string *blocks* of length $m/\beta$. We obtain strings $x_0,x_1,\ldots ,x_{2\beta -1}$, such that $x_i=x'[\frac{im}{\beta } \mathinner {.\,.}\frac{(i+1)m}{\beta }-1]$, for all $0 \le i < 2\beta$. We split *y* in $\beta$ non-overlapping string blocks of length $n/\beta$. We obtain strings $y_0,y_1,\ldots ,y_{\beta -1}$, such that $y_i=y[\frac{in}{\beta } \mathinner {.\,.}\frac{(i+1)n}{\beta }-1]$, for all $0 \le i < \beta$.

**Step 2**: For a given sequence $x_j,\ldots ,x_{j+{\beta -1}}$ of strings and *y*, we compute the $\beta$-blockwise *q*-gram distance as follows

$$\begin{aligned} \delta _j=D_{\beta ,q}\left(x'\left[\frac{jm}{\beta } \mathinner {.\,.}\frac{jm}{\beta }+m-1\right], y \right)=\sum \limits _{i=0}^{\beta -1} D_{q}(x_{j+i}, y_i). \end{aligned}$$

We compute $\delta _j$, for all $0 \le j \le \beta$. We choose $j_{\textit{best}}=j$ such that $\delta _j$ is minimal, for all $0 \le j \le \beta$. In other words, we have found a *window* of length *m* starting at position $j_{\textit{best}}$, such that $(j_{\textit{best}}+1) \bmod (m/\beta )=0$, consisting of $\beta$ blocks of length $m/\beta$ each, that minimizes its $\beta$-blockwise *q*-gram distance from *y*.

**Step 3**: To perform a refinement on the position of the window, we consider all starting positions included in the two blocks starting at positions $j_{\textit{best}}$ and $j_{\textit{best}} - m/\beta$. This includes $2m/\beta - 1$ starting positions in total—we do not need to consider position $j_{\textit{best}} - m/\beta$ as this was already considered by another window in Step 2. Similarly to Step 2, we obtain the $\beta$-blockwise *q*-gram distance $\delta _i$ between the window starting at position *i* and *y*, for all $j_{\textit{best}} - m/\beta < i \le j_{\textit{best}} + m/\beta -1$. We report position $i_{\textit{best}} = i$ such that $\delta _i$ is minimal, for all $j_{\textit{best}} - m/\beta < i \le j_{\textit{best}} + m/\beta -1$.

**Analysis** Step 1 can be done trivially in time $\mathcal {O}(m + n)$. If we have space $\mathcal {O}(|\Sigma |^q)$ available, then, by Lemma 1, $D_{q}(x_{j+i}, y_i)$ can be computed in time $\mathcal {O}\left(\frac{m+n}{\beta }\right)$. By Lemma 2, $\delta _j$ can be computed in time $\mathcal {O}\left(\beta (\frac{m+n}{\beta })\right)=\mathcal {O}(m+n)$. Hence, Step 2 can be done in time $\mathcal {O}(\beta (m+n))$. In Step 3, the blockwise *q*-gram distance $\delta _i$ between a single window and *y* can be computed in time $\mathcal {O}\left(\beta (\frac{m+n}{\beta })\right)=\mathcal {O}(m+n)$. There exist $2m/\beta - 1$ such windows. Hence, Step 3 can be done in time $\mathcal {O}\left(\frac{m(m+n)}{\beta }\right)$. Overall, the algorithm requires time $\mathcal {O}\left(\beta (m+n) + \frac{m(m+n)}{\beta }\right)$ and space $\mathcal {O}(|\Sigma |^q + m + n)$.

For practical purposes, setting $\beta = \mathcal {O}(\sqrt{m})$ and $q = \mathcal {O}(\log _{|\Sigma |} m)$ gives an algorithm with time complexity $\mathcal {O}(\sqrt{m}(m + n))$ and space complexity $\mathcal {O}(m + n)$.

### Algorithm saCSC: an exact suffix-array-based algorithm

The above heuristic hCSC does not guarantee to find the exact value *i*, for which $\delta _{i}=D_{\beta ,q}(x^i, y)$ is minimal. In particular, when we identify $j_{\textit{best}}$ in Step 2, that is, the *j* for which $\delta _j$ is minimal, we take into account only the values of *j* such that ${(j+1) \bmod (m/\beta )}=0$. Thus, Step 3 cannot guarantee that $i_{\textit{best}}$, the local minimum obtained by shifting the window $m/\beta$ positions to the right and left of $j_{\textit{best}}$, is minimal for all $0\le i < m$. In this section, we give a fast and exact algorithm, denoted by saCSC, to find *i* such that $\delta _{i}=D_{\beta ,q}(x^i, y)$ is minimal, based on the suffix array (see "Definitions and properties" section).

We partially follow the idea from [37]. This work investigates the string matching problem in the setting of *k*-abelian equivalences: two strings are considered *k*-abelian equivalent for some positive integer *k*, if they have the same length and share the same factors of length at most *k*, including multiplicities. Note that if *k* is greater than or equal to the string’s length, then the strings must be equal. A version of this result, called extended *k*-abelian equivalence, focuses only on the factors of length *k*. By setting $k=q$, it is quite straightforward to notice the equivalence with *q*-grams. Therefore, in order to avoid confusion we will refer to the former notion from now on as *q-abelian equivalence*.

In [37], the authors propose a linear-time algorithm to solve the string matching problem when looking at *q*-abelian equivalent strings: given a string *x* of length *m*, a string *y* of length $n \ge m$, and a positive integer $q < m$, all factors of *y* that are *q*-abelian equivalent to *x* can be found in time and space $\mathcal {O}(m + n)$. The idea of the algorithm in [37] consists in constructing the suffix array of the string *xy*, and ranking sets of identical *q*-length prefixes of suffixes in the suffix array in the order of their appearance. Then it constructs new strings based on this ranking, and solves the problem as in the *jumbled matching* case [38], i.e. identifying all factors of *y* that have the same Parikh vector as *x*.

We first describe our algorithm for a single block ($\beta = 1$) and then address the general case ($\beta \ge 1$).

**Basic algorithm for**$\beta = 1$. We construct the suffix array of the string *xxy* and assign a *rank* to the prefix with length *q* of each suffix with length at least *q*, based on its order in the suffix array. That is, the first $i_0$ suffixes, of length at least *q*, in the suffix array, all sharing the same prefix of length *q*, will get rank 0; the next $i_1$ suffixes, of length at least *q*, sharing the same prefix of length *q*, different from the previous one, will get rank 1, and so on. Next, based on this ranking, we construct two new strings $x'$ of length $2m-q+1$ and $y'$ of length $n-q+1$, such that $x'[i]=j$, if *j* is the rank of the *q*-length prefix of the $(i+1)$th suffix of *xx* in the suffix array of *xxy* (the same goes for *y*). It is not difficult to see that the ranks go up at most to value $m+n-q+1$. However, we can reduce this value to $m+2$ by introducing two new ranks $a_x$ and $a_y$: we can conceptually replace by $a_x$ every letter of $x'$ that does not occur in $y'$, and by $a_y$ every letter of $y'$ that does not occur in $x'$. Hence we can consider that the new strings $x'$ and $y'$ are defined over an integer alphabet of size *at most*$\min (n-q+1, m)+2\le m+2$.

#### *Example 3*

Let $x=\texttt {GAGTCTA}$, $y=\texttt {TCTAGCG}$, and $q=3$. We denote *xxy* by *z*.

**Table:** 

*i*	0	1	2	3	4	5	6	7	8	9	10	11	12	13	14	15	16	17	18	19	20
*z*[*i*]	G	A	G	T	C	T	A	G	A	G	T	C	T	A	T	C	T	A	G	C	G
$\textsf {SA}[i]$	6	17	1	8	13	19	4	15	11	20	0	7	18	2	9	5	16	12	3	14	10
$\textsf {LCP}[i]$	0	2	2	6	1	0	1	4	3	0	1	7	1	1	5	0	3	2	1	5	4
$x'[i]$	$a_x$	$a_x$	$a_x$	2	0	1	$a_x$	$a_x$	$a_x$	$a_x$	2	0									
$y'[i]$	2	0	1	$a_y$	$a_y$																

Here, $x'[3] = y'[0] = 2$ denotes that $x[3 \mathinner {.\,.}5] = y[0 \mathinner {.\,.}2] = \texttt {TCT}$ and $x'[0] = a_x$ denotes that $x[0 \mathinner {.\,.}2] = \texttt {GAG}$ does not occur in *y*.

We observe that when identifying the *q*-gram distance between two blocks, we can apply the idea in [37], with the only difference that we should also maintain a Parikh vector that stores the *differences* between the number of occurrences of *q*-grams (in fact the new letters given by the ranks) in the current block of *xx* and *y*. Moreover, at the time of the construction of $y'$, we also construct a Parikh vector $\mathcal {P}(y')$, storing for each letter of $y'$, the number of its occurrences in $y'$. Notice that $|\mathcal {P}(y')|\le m+2$. Later on, when computing the *q*-gram distances, we can construct another vector $\textsf {diff}$ to store the letter differences between $\mathcal {P}(y')$ and the Parikh vector covering the $m - q + 1$ letters of $x'$ associated with a window of length *m* on the string *xx*. This gives us the current Parikh difference and, in fact, represents the *q*-gram distance between the two analyzed blocks, where $|\textsf {diff}|\le m+2$. Apart from these, we only need another vector $\delta$ of size *m*, which stores at each position *i* the actual *q*-gram distance $\delta _i$ between *y* and the window starting at position *i* in *xx*, which is the *i*th rotation $x^i$ of *x*.

We use a sliding window of length *m* to maintain the above information. When the window is shifted one position to the right, we have to add to the difference-vector $\textsf {diff}$ the previous first element of the window, and deduct from it the current last element of it. The distance $\delta _i$ between $y'$ and the factor of $x'$ starting at position *i* is thus updated using, in addition, the value of the *q*-gram distance $\delta _{i-1}$ as follows. If, after adding the previous first element to the vector, we have a non-positive value at this position, we update the distance by decreasing the previous value by 1; otherwise, we increase it by 1. If, after deducting the current last element to the vector, we have a non-negative value at this position, we update the distance by decreasing the previous value by 1; otherwise, we increase it by 1. The distance will never be less than the number of occurrences of $a_y$. Furthermore, if the previous first element was $a_x$, the new distance decreases by 1, and for every newly added $a_x$, it increases by 1. As these operations require constant time, after going once through $x'$ with $y'$, we obtain the list of distances $\delta _i$ from *y* to each rotation $x^i$ in linear time.

We are now able to give a more formal description of the steps to solve the CSC problem for $\beta = 1$, which follow a dynamic programming scheme.

**Step 1**: Construct the SA, iSA, and LCP of *xxy*. Rank the *q*-length prefixes of suffixes using $\textsf {LCP}$-array queries. Construct $x'$ and $y'$, as well as $\mathcal {P}(y')$, the Parikh vector storing, for each letter of $y'$, the number of its occurrences in $y'$; making proper use of letters $a_x$ and $a_y$, the ranks that do not occur in either $y'$ or $x'$, respectively. Further, create $\textsf {diff}=\mathcal {P}(y')$ and $\delta _0=\sum _{i=0}^{|\mathcal {P}(y')|-1} \mathcal {P}(y')[i].$

#### *Example 4*

Following Example 3, let $x=\texttt {GAGTCTA}$, $y=\texttt {TCTAGCG}$, $q=3$, and $z=xxy$.

**Table:** 

*i*	0	1	2	3	4	5	6	7	8	9	10	11	12	13	14	15	16	17	18	19	20
*z*[*i*]	G	A	G	T	C	T	A	G	A	G	T	C	T	A	T	C	T	A	G	C	G
$x'[i]$	$a_x$	$a_x$	$a_x$	2	0	1	$a_x$	$a_x$	$a_x$	$a_x$	2	0									
$y'[i]$	2	0	1	$a_y$	$a_y$																

The table below represents vector $\textsf {diff}$, right after the execution of Step 1, which implies that $\delta _0 = 5$.

**Table:** 

$a_x$	0
$a_y$	2
0	1
1	1
2	1

**Step 2**: Read the first $m-q+1$ letters of $x'$, which constitute our sliding window of length *m* on the string *xx*. When reading letter $x'[i]$, update $\textsf {diff}$ by decreasing by 1 the value of the newly read letter, and update $\delta _0$, by either increasing the current value of the distance when there were read too many of the current letters, or decreasing it, when more of these letters still occur in $y'$

$$\begin{aligned} \textsf {diff}[x'[i]] = \textsf {diff}[x'[i]]-1 \, \text{ and }\quad \delta _0 = \left\{ \begin{array}{ll} \delta _0 - 1, \quad &{}\mathrm {if }\ \textsf {diff}[x'[i]] \ge 0\\ \delta _0 + 1, \quad &{}\mathrm {if }\ \textsf {diff}[x'[i]] < 0. \end{array} \right. \end{aligned}$$

#### *Example 5*

Following Example 4, the table below represents vector $\textsf {diff}$, right after the execution of Step 2, which implies that $\delta _0 = 6$.

**Table:** 

$a_x$	3
$a_y$	2
0	0
1	1
2	0

**Step 3**: Let *i* be the current position in $x'$ and repeat this step, one position at a time. Shift the window to the right, update the information for $\textsf {diff}$

$$\begin{aligned} \textsf {diff}[x'[i]] = \textsf {diff}[x'[i]]+1 \,\text{ and }\quad \textsf {diff}[x'[i+m]] = \textsf {diff}[x'[i+m]]-1, \end{aligned}$$

and calculate $\delta _{i+1}$, based on this information, sequentially applying the two following rules

$$\begin{aligned} \delta _{i+1} = \left\{ \begin{array}{ll} \delta _i - 1, \quad &{}\mathrm {if }\ \textsf {diff}[x'[i]] \le 0\\ \delta _i + 1, \quad &{}\mathrm {if }\ \textsf {diff}[x'[i]] > 0 \end{array} \right. \end{aligned}$$

$$\begin{aligned} \delta _{i+1} = \left\{ \begin{array}{ll} \delta _{i+1} - 1, \quad &{}\mathrm {if }\ \textsf {diff}[x'[i+m]] \ge 0\\ \delta _{i+1} + 1, \quad &{}\mathrm {if }\ \textsf {diff}[x'[i+m]] < 0. \end{array} \right. \end{aligned}$$

#### *Example 6*

Following Example 5, the table below represents vector $\textsf {diff}$ at iteration $i'=3$ of Step 3, which implies that $\delta _0 = 4$. This is in fact the best rotation of *x*, that is, $x^3 = \texttt {TCTAGAG}$.

**Table:** 

$a_x$	2
$a_y$	2
0	0
1	0
2	0

**Correctness** Steps 1 and 2 are trivially correct as at the end of them we have that $\textsf {diff}$ is the difference between $\mathcal {P}(y')$ and the vector corresponding to the window. These operations follow directly from the definitions of $\textsf {SA}$ and $\textsf {LCP}$, and are followed by a simple traversal of the suffix array in order to obtain the ranks and create the $\mathcal {P}(y')$ and $\textsf {diff}$ vectors. Also, $\delta _0$, which was initially the number of letters in $y'$, is decreasing as long as the difference between the vectors for a specific letter is non-negative (thus, we still have more occurrences of that letter in $y'$ compared to the window), and increasing otherwise. In Step 3, we update the difference vector by increasing the value at position $x'[i]$ and decreasing that of the new letter $x'[i+m]$ added to the difference. The *q*-gram distance at that position is based on the values of the newly obtained difference vector, as well as the *q*-gram distance at the previous position: if $\textsf {diff}[x'[i]] \le 0$, then obviously there were more letters $x'[i]$ in $y'$ than in the window, thus we need to decrease, while, if $\textsf {diff}[x'[i]] > 0$, then there were at least as many letters $x'[i]$ in the window as in $y'$, and taking one out increases the distance. The complementary reasoning applies to the newly added letter $x'[i+m]$. The value of $\delta _i$ never goes below the number of occurrences of $a_y$ in $y'$ (it is equal to that, when all other elements of $\textsf {diff}$ are 0) and represents the *q*-gram distance between *y* and $x^i$, the corresponding window of length *m* starting at position *i* in *xx*.

**Analysis** In Step 1, constructing SA, iSA, and LCP of *xxy* can be done in time and extra space $\mathcal {O}(m+n)$ ("Definitions and properties" section). Furthermore, the construction of $x'$, $y'$, $\mathcal {P}(y')$, $\textsf {diff}$, and $\delta _0$ is done with the same time and space cost. In Step 2, updating $\textsf {diff}$ and $\delta _0$ after reading each letter takes constant time, as we execute two operations, thus $\mathcal {O}(m)$ in total. Constant time is required for each iteration in Step 3 to compute the value of $\delta _i$, $1 \le i < m$, and update $\textsf {diff}$, since a constant number of operations are executed, thus $\mathcal {O}(m)$ in total. Hence, we can solve the CSC problem for $\beta =1$ in time and space $\mathcal {O}(m+n)$.

**General algorithm for**$\beta \ge 1$. We can now generalize this algorithm to solve the CSC problem for any $\beta \ge 1$, which gives algorithm saCSC. We maintain a Parikh vector for each block, and apply the above basic algorithm for the *j*th block in each string, computing their *q*-gram distance. If we denote by $\mathcal {P}_j(y')$ and $\textsf {diff}_j$, for all $0\le j<\beta$, the $\beta$ Parikh vectors of $y'$ and of the *q*-gram distances, respectively, as well as by $\delta _{i,j}$ the *q*-gram distance between the *j*th block of *y* and $x^i$, then the updates will be given by the formulae below. Hence, at each position $i<m$, we can update all of the $\beta$ Parikh vectors corresponding to the blocks, as previously described, in time $\mathcal {O}(\beta )$. As an example, see here the modification of the previous Step 3, with the other two steps being easily adapted in a similar fashion.

**Step 3’:** When shifting the window one position to the right from position *i*, update the information for every $\textsf {diff}_j$, where $0\le j< \beta$, as follows

$$\begin{aligned} \textsf {diff}_j\left[x'\left[i+\frac{jm}{\beta }\right]\right]&= \textsf {diff}_j\left[x'\left[i+\frac{jm}{\beta }\right]\right]+1\\ \textsf {diff}_j\left[x'\left[i+\frac{(j+1)m}{\beta }\right]\right]&= \textsf {diff}_j\left[x'\left[i+\frac{(j+1)m}{\beta }\right]\right]-1, \end{aligned}$$

and calculate $\delta _{i+1,j}$, based on this information, sequentially applying the two following rules

$$\begin{aligned} \delta _{i+1,j} = \left\{ \begin{array}{ll} \delta _{i,j} - 1, \quad &{}\mathrm {if }\ \textsf {diff}_j \left[x'[i+\frac{jm}{\beta }] \right] \le 0\\ \delta _{i,j} + 1, \quad &{}\mathrm {if }\ \textsf {diff}_j \left[x'[i+\frac{jm}{\beta }] \right] > 0 \end{array} \right. \end{aligned}$$

$$\begin{aligned} \delta _{i+1,j} = \left\{ \begin{array}{ll} \delta _{i+1,j} - 1, \quad &{}\mathrm {if }\ \textsf {diff}_j \left[x'[i+\frac{(j+1)m}{\beta }]\right] \ge 0\\ \delta _{i+1,j} + 1, \quad &{}\mathrm {if }\ \textsf {diff}_j \left[x'[i+\frac{(j+1)m}{\beta }]\right] < 0. \end{array} \right. \end{aligned}$$

Therefore, we obtain the following result.

#### **Theorem 1**

*Algorithm *s solves the CSC problem in aCSC$\mathcal {O}(\beta m + n)$* time and space.*

## Implementation

We implemented algorithms nCSC, hCSC, and saCSC as the program CSC. Given one of the three methods, two sequences *x* and *y* in (Multi)FASTA format, the number $\beta$ of blocks, and the length *q* of the *q*-grams, CSC finds the rotation of *x* (or an approximation of it) that minimizes its $\beta$-blockwise *q*-gram distance from *y*. The implementation is distributed under the GNU General Public License (GPL), and it is available freely at http://www.github.com/solonas13/csc.

For comparison purposes, we implemented a naïve algorithm that compares all rotations of *x* against *y* using the Needleman-Wunsch algorithm [39] with substitution matrices and affine gap penalty scores [40]; we denote this implementation by cNW. We also implemented the following heuristics. We first use the Smith-Waterman local alignment algorithm [41] to search for the best local alignment of *x* and *y* and then use a central match from this local alignment to anchor the global alignment (see also [11]); we denote this implementation by hSW.

### Refining algorithm saCSC

The application of the $\beta$-blockwise *q*-gram distance via algorithm saCSC suggests that an optimal or a close-to-optimal rotation of *x* can be found when compared to cNW. Due to the locality property offered by the newly introduced distance notion, it is reasonable to assume that the close-to-optimal rotation returned by saCSC may be refined via some quick heuristics that take into consideration the blocks at both ends.

Let $x^i$ be the close-to-optimal rotation of *x* returned by saCSC. We introduce a new input parameter $0 < p \le \tfrac{\beta }{3}$, which defines the length L of the prefixes and suffixes of $x^i$ and *y* to be considered in the refinement as follows:

$$L = \left\lfloor {p \times \frac{m}{\beta }} \right\rfloor.$$

We take *p* block(s) of the *prefix* of $$x^i$$, concatenate it with a string of equal length *L* comprised only of letter $, where $$\$ \notin \Sigma$$, and concatenate that with *p* block(s) of the *suffix* of $$x^i$$ to form a new string $$x''$$ of length 3*L*. We do the same with *y* to form a new string $$y''$$.

The refinement algorithm works by taking all rotations of $$x''$$ and comparing their similarity to $$y''$$. Each rotation of $$x''$$ is compared to $$y''$$ excluding when a $ letter is found at index 0 of the rotation of $$x''$$. We measure the similarity between the strings for which equality between letters are positively valued; inequalities, insertions, and deletions are negatively valued; and comparisons involving $ are neither positively nor negatively valued. The goal of rotating $$x''$$ serves to find the rotation that maximizes the similarity to $$y''$$ and, to this end, we make use of the Needleman-Wunsch algorithm. The rotation of $$x''$$ which results in the maximum score is chosen as the best rotation, and hence, the final rotation $$x^{i'}$$ of *x* is computed based on this rotation of $$x''$$. Ties are broken arbitrarily. We denote this new algorithm, consisting of saCSC and the refinement stage, by saCSCr.

The application of the Needleman-Wunsch algorithm on strings of length 3*L* has a time complexity of $\mathcal {O}(L^2)$. Considering all rotations of $x''$ results in a time complexity of $\mathcal {O}(L^3)$ for the refinement step. Overall, saCSCr takes time $\mathcal {O}(\beta m + n + L^3)$.

#### Example 7

Consider the following pair of strings obtained from saCSC

$$\begin{aligned}&x^i = \texttt {GACACCCCCCACAGTTTATGTAGCTT\ldots ACCCCGAACCAACCAAACCCCAAA}\\&y = \texttt {GTTTATGTAGCTTACCTCCCCAAAGC\ldots CAAACCCCAAAGACACCCCACACA} \end{aligned}$$

and the following pair of strings formed for $L = 25$.

$$\begin{aligned}&x'' = \texttt {GACACCCCCCACAGTTTATGTAGCTT\$\$\$\$\$\$\$\$\$\$\$\$\$\$\$\$\$\$\$\$\$\$\$\$\$ACCCCGAACCAACCAAACCCCAAA}\\&y'' = \texttt {GTTTATGTAGCTTACCTCCCCAAAG\$\$\$\$\$\$\$\$\$\$\$\$\$\$\$\$\$\$\$\$\$\$\$\$\$CAAACCCCAAAGACACCCCACACA} \end{aligned}$$

The Needleman-Wunsch algorithm for all rotations of $x''$ and string $y''$ gives the following optimal alignment

$$\begin{aligned}&\texttt {GTTTATGTAGCTT\$\$\$\$\$\$\$\$\$\$\$\$\$\$\$\$\$\$\$\$\$\$\$\$\$ACCCCGAACCAACCAAACCCCAAAGACACCCCCCACA}\\&\texttt {GTTTATGTAGCTTACCTCCCCAAAG\$\$\$\$\$\$\$\$\$\$\$\$\$\$\$\$\$\$\$\$\$\$\$\$\$CAAACCCCAAAGACACCCCACACA} \end{aligned}$$

which tells us that a refined rotation is in fact $x^{i'}$, where $i' = i - 13$

$$\begin{aligned}&x^{i'} = \texttt {GTTTATGTAGCTT\ldots CAAACCCCAAAGACACCCCCCACA}\\&y = \texttt {GTTTATGTAGCTT\ldots CAAACCCCAAAGACACCCCACACA} \end{aligned}$$

## Experimental results

The following experiments were conducted on a desktop computer using one core of Intel^®^ Core^TM^ i7-2600 CPU at 3.4GHz and 12GB of RAM under 64-bit GNU/Linux. All programs were compiled with gcc version 4.7.3. We used both synthetic data and real data. All input datasets referred to in this section are publicly maintained at the same web-site. First, in "Accuracy"–"Time performance" sections, we establish the quality (accuracy and performance) of our methods. Then, in "Application to synthetic data"–"Application to real data" sections, we show applications of our methods.

### Accuracy

We began with simulating three DNA sequence datasets using INDELible [42], with each dataset consisting of 12 sequences (denoted by $\alpha$), each of length approximately 2500 bp (denoted by $\gamma$). INDELible produces linear sequences with substitutions, insertions, and deletions at rates defined by the user. Three unique substitution rates (denoted by $\theta$) were set, per dataset, using the substitution model JC69 (Jukes-Cantor, 69): 5, 20, and 35 %. The insertion and deletion rates were set, respectively, to 4 and 6 % (denoted by $\kappa$ and $\omega$), relative to substitution rate of one, similar to those observed in MtDNA in primates and mammals [25]. We refer to these datasets as *Original*.

**Fig. 1:**
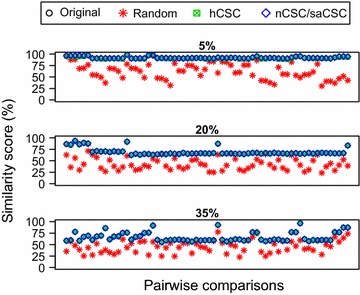
Accuracy. Accuracy comparison for substitution rates 5, 20, and 35 %; the* black*,* green*, and* blue points* coincide implying that algorithms hCSC, nCSC, and saCSC return the rotation maximizing the similarity score for all pairwise comparisons

To allow for comparison of the performance of the algorithms in realigning randomly rotated sequences, which should be similar to those obtained from sequencing circular DNA structures, such as MtDNA, one random rotation was generated in each sequence in all datasets, creating new datasets which will be referred to as *Random*. Using the three *Random* datasets allowed us to test the accuracy of hCSC and saCSC; notice that nCSC and saCSC always return the same rotation. For each *Random* dataset, an all-against-all sequence comparison was performed. That is, all 66 possible pairs of sequences in each dataset were given as input to both hCSC and saCSC. $\beta$ was set to $\lceil \sqrt{m} \rceil =50$ and *q* was set to $\lceil \log _{|\Sigma |} m \rceil =6$. The resultant re-rotated sequences were aligned using EMBOSS Needle (default parameters) and the similarity scores were compared to those of the *Original* and *Random* datasets, which were input directly to EMBOSS Needle (default parameters). The results can be found in Fig. 1.

The results show that: (a) hCSC and saCSC yield significantly improved similarity scores compared to those obtained from giving *Random* datasets as input directly to EMBOSS Needle; and (b) hCSC and saCSC yield similarity scores that are identical or almost identical—notice that the black (Original), green (hCSC), and blue (nCSC/saCSC) points *coincide*—to those obtained from giving *Original* datasets as input directly to EMBOSS Needle. This implies that algorithms hCSC, nCSC, and saCSC return the rotation maximizing the similarity score for all pairwise comparisons.

Hence, we establish here that the introduced distance measure coupled with the respective algorithms consistently yield a very high accuracy, compared to the standard measure [17, 39, 40], for both *low* and *high* substitution rates.

### Time performance

We then compared the time performance of the algorithms. Each algorithm was given a pair of randomly generated sequences starting from $m=n=50$ bp and doubling 8 times to a length of $m=n=12,800$ bp. It was expected that the slowest algorithm would be cNW which runs in time $\mathcal {O}(nm^2)$. Then it would be algorithm nCSC which runs in time $\mathcal {O}(m(m + n))$, then algorithm hCSC, which runs in time $\mathcal {O}\left (\beta (m+n) + \frac{m(m+n)}{\beta } \right)$, and lastly algorithm saCSC, which runs in time $\mathcal {O}({\beta }m + n)$.

**Fig. 2:**
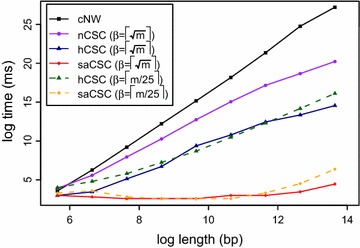
Time performance. Elapsed-time comparison of algorithms cNW, nCSC, hCSC, and saCSC

Initially, $\beta$ was set to $\lceil \sqrt{m}\rceil$ and *q* was set to $\lceil \log _{|\Sigma |} m \rceil$. The results in Fig. 2 demonstrate orders-of-magnitude superiority of saCSC compared to cNW and nCSC, confirming our theoretical findings. Algorithm hCSC is the second fastest. Although $\beta$ was set to $\lceil \sqrt{m}\rceil$, saCSC clearly outperforms hCSC, due to the use of a highly optimized implementation of the suffix-array construction [43], thus highlighting the importance of suitably implemented data structures such as suffix arrays.

Since the time complexities of hCSC and saCSC depend on $\beta$, we repeated the same experiment with these two algorithms setting $\beta$ to $\lceil m/25 \rceil$ and *q* to $\lceil \log _{|\Sigma |} m \rceil$—notice that *q* does not affect the time efficiency of the algorithms. The results in Fig. 2 show that hCSC and saCSC are still the fastest, even though $m=\mathcal {O}(\beta )$, and that saCSC is clearly the fastest of all. As expected for $m=\mathcal {O}(\beta )$, we observe that hCSC and saCSC become gradually slower as *m* grows.

More algorithms could have been included in the comparison but their (at least) quadratic time complexity [22, 23] prevents them from competing with saCSC.

### Application to synthetic data

For evaluating the proposed methods for circular sequence comparison in some relevant application, we also implemented the following pipeline for distance-based phylogenetic reconstruction of a dataset with *N* circular sequences.

1. For each pair (*x*, *y*) of the *N* sequences, we use one method for circular sequence comparison to compute the best rotation $x^i$.
2. A *similarity* score for $(x^i,y)$ is then computed using EMBOSS Needle (default parameters) and stored in cell [*x*, *y*] of an $N\times N$ similarity score matrix.
3. The similarity score matrix is transformed into a distance matrix by converting each score into a *distance* relative to the maximum score in the similarity score matrix.
4. Neighbour joining clustering is performed on the distance matrix, using NINJA [44], to produce a phylogenetic tree.

Phylogenetic trees were constructed by NINJA [44], for the aforementioned *Random* datasets, using output from the following algorithms: cNW (EMBOSS default parameters), hSW (see introduction of "Implementation" section, EMBOSS default parameters), and saCSCr ($\beta =50$, $q=5$, and $p = 1$). Notice that, output from cNW should be the same as from EMBOSS Needle (default parameters) with the *Original* datasets as input. In terms of accuracy, the Robinson-Foulds (RF) distance metric [45, 46] was used to compare the three resultant phylogenetic trees with the tree resulting from EMBOSS Needle (default parameters) on the *Original* and *Random* datasets, denoted by NW(o) and NW(r), respectively. RF distance can be defined as the number of operations required to transform one tree in to another. If two isomorphic trees share the same labelling then they have an RF distance of 0. The results displayed in Table 3 clearly show that saCSCr and cNW produce the most accurate results with these nine datasets. As also shown in [11], hSW followed by EMBOSS Needle (default parameters) can often result in sub-optimal global alignments.

**Table 3:** RF distances between the tree obtained from the NW(o) and those obtained from NW(r), cNW, hSW, and saCSCr

Dataset $<\alpha ,\gamma ,\theta ,\kappa ,\omega>$	NW(r)	cNW	hSW	saCSCr
<12, 2500, 0.05, 0.06, 0.04>	16	0	0	0
<12, 2500, 0.20, 0.06, 0.04>	12	0	0	0
<12, 2500, 0.35, 0.06, 0.04>	4	0	0	0
<25, 2500, 0.05, 0.06, 0.04>	44	0	0	0
<25, 2500, 0.20, 0.06, 0.04>	24	0	0	0
<25, 2500, 0.35, 0.06, 0.04>	16	0	0	0
<50, 2500, 0.05, 0.06, 0.04>	86	0	6	0
<50, 2500, 0.20, 0.06, 0.04>	84	0	0	0
<50, 2500, 0.35, 0.06, 0.04>	56	0	0	0

The number of sequences in the dataset is denoted by $\alpha$; $\gamma$ denotes their lengths; $\theta$ denotes the substitution rate; $\kappa$ and $\omega$ denote the relative insertion and deletion rates, respectively

In terms of time performance, the elapsed time required for each method to process each dataset was recorded and the results are displayed in Table 4. It is clear, from the results presented heretofore, that saCSCr outperforms all other algorithms by at least one order of magnitude.

**Table 4:** Elapsed-time comparison (in seconds) for algorithms cNW, hSW, and saCSCr

Dataset $<\alpha ,\gamma ,\theta ,\kappa ,\omega>$	cNW	hSW	saCSCr
<12, 2500, 0.05, 0.06, 0.04>	10,139.36	72.43	6.90
<12, 2500, 0.20, 0.06, 0.04>	9888.84	80.91	6.57
<12, 2500, 0.35, 0.06, 0.04>	10,052.33	80.16	6.28
<25, 2500, 0.05, 0.06, 0.04>	46,311.85	369.02	27.61
<25, 2500, 0.20, 0.06, 0.04>	46,230.07	375.41	28.92
<25, 2500, 0.35, 0.06, 0.04>	46,289.99	400.30	30.44
<50, 2500, 0.05, 0.06, 0.04>	122,165.95	1563.96	125.63
<50, 2500, 0.20, 0.06, 0.04>	121,810.69	1617.89	123.12
<50, 2500, 0.35, 0.06, 0.04>	120,679.32	1662.82	123.77

The number of sequences in the dataset is denoted by $\alpha$; $\gamma$ denotes their lengths; $\theta$ denotes the substitution rate; $\kappa$ and $\omega$ denote the relative insertion and deletion rates, respectively

### Application to real data

We have concluded thus far that using $\beta = \lceil \sqrt{m} \rceil$ and $q = \lceil \log _{|\Sigma |} m \rceil$ results in a reasonable trade-off between running time and accuracy. In the following section, where necessary, we adopt these values and multiply or divide them by a constant factor (factor of two), depending on the length of the input sequences.

#### DNA sequences

**Pairwise sequence comparison.** As the input dataset, we used two real sequences from GenBank: human (NC_001807) and chimpanzee (NC_001643) MtDNA sequences. The MtDNA genome size for human is 16,571 bp and for chimpanzee is 16,554 bp. Their pairwise sequence alignment using EMBOSS Needle (default parameters) gives a similarity of $85.1\,\%$. We used cNW (EMBOSS default parameters) to obtain the rotation of NC_001807 that maximizes its similarity score with NC_001643. This experiment took approximately 28 h and the resultant rotation 578 of NC_001807 improved the similarity score to $91\,\%$. This result was then compared to those obtained from saCSC (equivalent to saCSCr with $p=0$) and saCSCr with varying parameters, displayed in Table 5.

**Table 5:** Rotations of GenBank sequence NC_001807 obtained when compared to NC_001643 with varying parameters of saCSCr

*q*	$\beta$	*p*	Rotation
5	50	0	566
5	50	1	*578*
5	$\sqrt{m}$	0	567
5	$\sqrt{m}$	1	*578*
5	$2\sqrt{m}$	0	583
5	$2\sqrt{m}$	1	*578*
5	$\frac{\sqrt{m}}{2}$	0	566
5	$\frac{\sqrt{m}}{2}$	1	*578*

The convergence of the results after the additional step of refinement (see Table 5 in italics) demonstrates the convenience and necessity of saCSCr.

For clarity of presentation hereafter, instead of using $\beta$, we denote by $\ell$ the length of the block chosen in algorithm saCSCr.

We repeated this experiment with the human and gorilla (NC_011120) MtDNA sequences. The MtDNA genome size for gorilla is 16,412 bp. Their pairwise sequence alignment using EMBOSS Needle (default parameters) gives a similarity of $83.5\,\%$. After using saCSCr to rotate sequence NC_001807 ($\ell =50$, $q=5$, and $p = 1$), EMBOSS Needle (default parameters) gave a significantly improved similarity of $88.4\,\%$.

Finally, note that the experiments which used saCSC and saCSCr each took a fraction of a second to run.

**Distance-based phylogenetic reconstruction** Three datasets of 16 primate, 12 mammalian and 19 mixed mammalian and primate MtDNA sequences, of average length 16,500 bp, were obtained from GenBank. We followed the same pipeline as described in "Application to synthetic data" section. The RF distance between the trees produced by cNW (EMBOSS default parameters), and the trees produced by saCSCr ($\ell = \lceil \sqrt{m} \rceil = 129$, $q = 5$, and $p=1$) followed by EMBOSS Needle (default parameters), was 0.

#### RNA sequences

Eighteen viroid sequences were obtained from RefSeq, a database of curated molecular biological sequences [47]. Their lengths and target hosts vary, ranging from 348 to 371 bp and infecting peppers and citrus fruits, respectively. We followed the same pipeline as described in "Application to synthetic data" section. The RF distance between the tree produced by cNW (EMBOSS default parameters), and the tree produced by saCSCr ($\ell = \lceil \sqrt{m} \rceil =19$, $q= \lceil \log _{|\Sigma |} m \rceil =5$, and $p=1$) followed by EMBOSS Needle (default parameters), was 0.

#### Protein sequences

**Linear, circularly-permuted protein sequences** Eight sequences of proteins, of average length 950 amino acids, belonging to $\beta$-glucosidase family [48] were obtained from the UniProt protein database [49]. We followed the same pipeline as described in "Application to synthetic data" section. The RF distance between the tree produced by cNW (EMBOSS default parameters), and the tree produced by saCSCr ($\ell = \lceil \sqrt{m} \rceil =31$, $q= \lceil \log _{|\Sigma |} m \rceil =5$, and $p=1$) followed by EMBOSS Needle (default parameters), was 0.

**Naturally-occurring circular proteins** Ten bacteriocin protein sequences, of average length 20 amino acids, were obtained from Cybase [50], a database of cyclical protein sequences. We followed the same pipeline as described in "Application to synthetic data" section. The RF distance between the tree produced by cNW (EMBOSS default parameters), and the tree produced by saCSCr ($\ell = 2 \lceil \sqrt{m} \rceil = 10$, $q= 2 \lceil \log _{|\Sigma |} m \rceil = 6$, and $p=1$) followed by EMBOSS Needle (default parameters), was 0.

## Conclusions

In this paper, we introduced a new distance measure for sequence comparison based on *q*-grams, and showed how it can be applied *effectively* and computed *efficiently* for circular sequence comparison. The most efficient algorithm presented here, saCSC, solves our defined problem CSC, exactly. Extensive experimental results, using both real and synthetic data, show that it maintains an accuracy very competitive to the optimal obtained after considering all rotations of *x* against *y* naïvely using global alignments. We also showed that algorithm saCSCr can bridge the gap between the optimal solution and our approximation via an additional refinement step. Finally, the presented experimental study demonstrates orders-of-magnitude superiority of our approach in terms of runtime efficiency. Our immediate target is to implement algorithm saCSCr in BEAR [24], a state-of-the-art tool for improving multiple circular sequence alignment.
