# Erratum to: Circular sequence comparison: algorithms and applications

**DOI:** 10.1186/s13015-016-0084-6

**Published:** 2016-07-27

**Authors:** Roberto Grossi, Costas S. Iliopoulos, Robert Mercas, Nadia Pisanti, Solon P. Pissis, Ahmad Retha, Fatima Vayani

**Affiliations:** 1 Department of Informatics, University of Pisa, Pisa, Italy; 2 ERABLE team, INRA, Paris, France; 3 Department of Informatics, King’s College London, London, UK; 4 Department of Computer Science, Kiel University, Kiel, Germany

## Erratum to: Algorithms Mol Biol (2016) 11:12 DOI 10.1186/s13015-016-0076-6

During the production process for this article [1], Theorem 1 in the ‘Algorithms’ section and Example 7 in the ‘Implementation’ section were formatted incorrectly. These have been updated and the corrected versions can now be found in the original article [1].
